# Target therapy of multiple myeloma by PTX-NPs and ABCG2 antibody in a mouse xenograft model

**DOI:** 10.18632/oncotarget.4663

**Published:** 2015-07-15

**Authors:** Cuiping Yang, Fei Xiong, Jun Dou, Jun Xue, Xi Zhan, Fangfang Shi, Miao Li, Songyan Wu, Shouhua Luo, Tianzhu Zhang, Yu Zhang, Ji Ming, Ning Gu

**Affiliations:** ^1^ Department of Pathogenic Biology and Immunology, School of Medicine & Collaborative Innovation Center of Suzhou NanoScience and Technology, Southeast University, Nanjing 210009, China; ^2^ School of Biological Science & Medical Engineering & Collaborative Innovation Center of Suzhou NanoScience and Technology, Southeast University, Nanjing 210096, China; ^3^ Department of Hematology, Affiliated Nanjing First Hospital, Nanjing Medical University, Nanjing 210006, China; ^4^ The Center for Vascular and Inflammatory Diseases, Department of Pathology, University of Maryland School of Medicine, Baltimore, MD 21201, USA; ^5^ Department of Oncology, Zhongda Hospital, Southeast University, Nanjing 210009, China

**Keywords:** multiple myeloma, cancer stem cells, paclitaxel, nanoparticles, ATP-binding cassette sub-family G member 2

## Abstract

Multiple myeloma (MM) remains to be an incurable disease. The purpose of this study was to evaluate the effect of ABCG2 monoclonal antibody (McAb) combined with paclitaxel (PTX) conjugated with Fe_3_O_4_ nanoparticles (NPs) on MM progressed from cancer stem cells (CSCs)in non-obese-diabetic/severe-combined-immunodeficiency (NOD/SCID) mouse model. Mice were injected with MM CSCs as marked by CD138^−^CD34^−^ phenotypes through tail veins. The developed MM mice were examined by micro-computer tomography scanning, ultrasonography and enzyme-linked immunosorbent analysis. These mice were then intravenously treated with different combinations of NPs, PTX, McAb, PTX-NPs and melphalan/prednisone once a week for four weeks. The injected mice developed characteristic MM-associated syndromes, including lytic bone lesions, renal damages and proteinuria. All the treated mice showed decrease in bone lesions, renal damages and anemia but increase in apoptosis compared with the mice treated with NPs only. In particular, the treatment with ABCG2 McAb plus PTX-NPs induced the strongest therapeutic response and had an efficacy even better than that of melphalan/prednisone, a conventional regimen for MM patients. These data suggest that PTX-NPs with ABCG2 McAb can be developed into potential treatment regimens for patients with relapsed/refractory MM.

## INTRODUCTION

Multiple myeloma (MM) is a debilitating disease characterized by neoplastic growth of plasma cells in the bone marrow and responsible for the most deaths of patients with hematologic malignancies. Clinically, MM patients often manifest destructive bone lesions, renal insufficiency, hypercalcemia and anemia. Agents of thalidomide, lenalidomide, bortezomib, melphalan/prednisone, and carfilzomib as well as hematopoietic stem cell transplantation have dramatically improved the therapeutic response and survival rate of MM patients. However, most MM patients are still facing a high risk of ultimate relapses due to the persistence of minimal residual malignant cells in the bone marrow. Consequently MM remains to be an incurable disease [1, 2]. Emerging evidence supports the view that a subpopulation of MM cells that are highly resistant to conventional cancer therapies shares certain features with cancer stem cells (CSCs) and may be responsible for tumor-initiating, self-renewal, drug resistances and metastasis [3–6]. Thus, it is clinically desirable to develop a therapeutic paradigm that targets specific molecules associated with MM CSCs, and thereby overcoming the drug resistance by eradicating CSCs.

It is known that CSCs often exhibit a high activity of ATP-binding cassette (ABC) transporter, in particular ABCG2 (ATP-binding cassette sub-family G member 2), a surface molecule that contributes to drug resistance by pumping out intracellular drugs. Indeed, there have been reports that using anti-ABC monoclonal antibodies are able to inhibit partially cellular drug resistance by compromising the growth of CSCs [7, 8].

Paclitaxel (PTX) has a broad spectrum of cytotoxicity towards a variety of cancers, including MM. However, it has a poor solubility, and clinical usage of its adjuvants such as Cremophor EL often causes severe allergic reactions, which significantly limits the efficacy of PTX-based therapies [6, 9]. Recent advances have evidenced that engineered nanoparticles (NPs) offer many therapeutic advances over traditional formulations because they can be readily manipulated into particles with desired sizes and surface properties that would make it possible to conjugate and deliver certain pharmaceutical compounds that are otherwise insoluble and unstable [10–15]. Magnetic nanoparticles (NPs) such as magnetite Fe_3_O_4_ based NPs have small sizes, large active surface areas, an excellent biocompatibility, and are readily internalized into cells and sustain well under endocytic environments. In the presented study we attempted to target MM CSCs by using a monoclonal antibody against ABCG2, and to deliver PTX by using NPs. To evaluate the anti-MM efficacy of the new approach, we also developed a MM model in which MM CSCs derived from either human MM RPMI 8266 cells or bone marrow mononuclear cells (BMMCs) from MM patients were used to develop MM-like malgiancies in xonografted mice. We found that application of NPs carrying PTX in combination with the ABCG2 antibody improved significantly the efficacy of inhibiting MM progression compared with that using each agent alone.

## RESULTS

### Mice injected with MM CSCs manifests typical MM-associated syndromes

**Figure 1:**
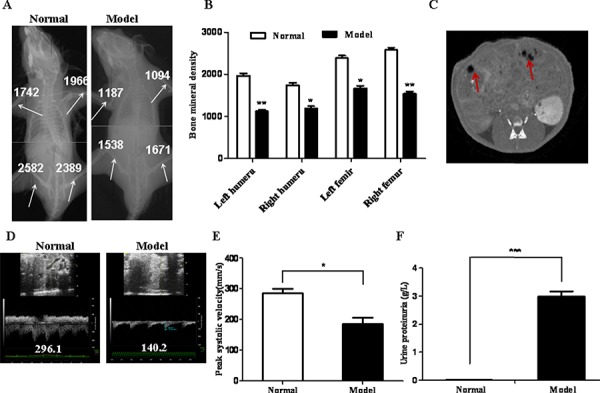
Establishment of a MM murine model **A.** Images showed Micro-CT scanning of a normal and a model mouse 18 days after injection of MM CD138^−^CD34^−^cells. BMD values at femur and humerus were indicated by arrows. **B.** Quantification of BMD as measured by Micro-CT (*n* = 6). **C.** A Micro-CT image of kidneys of a model mouse. Arrows indicate renal damaged areas. **D.** Ultrasound images showing peak systolic BFV of the renal artery of a normal and a model mouse. **E.** Quantification of peak systolic BFV. **F.** Urine protein was measured by ELISA. All the data represent mean ± S.D (*n* = 3). *p < 0.05, **p < 0.01 and ***p < 0.001 were calculated by *t* test, referring to the statistically significant difference as compared to the normal group.

To establish a MM animal model, we isolated MM CSCs from human MM RPMI 8226 cells based on the CD138^−^CD34^−^cell phenotypes^17^ and injected them via tail veins into NOD/SCID mice. Because MM patients were frequently associated with bone lesions and kidney damages, we examined on day 18 after implantation the bone marrow density (BMD) of humeri and femora of injected mice by micro-CT, a gold standard for imaging MM [16]. As shown in Fig. 1A and 1B, the BMD of both humerus and femurs of MM CSC-injected mice (model) was significantly reduced compared with the control group (normal). Micro-CT also detected a few low-density shadows (arrows, Fig. 1C) in the kidney of model mice, suggestive of the presence of MM lesions and renal impairment. Indeed, ultrasound imaging analysis of the renal arteries revealed that the peak systolic blood flow velocity (BFV) in the renal artery was significantly decreased in model mice (Fig. 1D and 1E). To assess further the renal damage, we measured protein contents in the urine and found that the proteinuria level was markedly enhanced in the model mice versus the normal mice (Fig. 1F). Thus, mice injected with MM CSCs shared many pathological characteristics with those of MM.

### PTX-NPs combined with ABCG2 McAb reduces effectively MM-associated bone lesions

**Figure 2:**
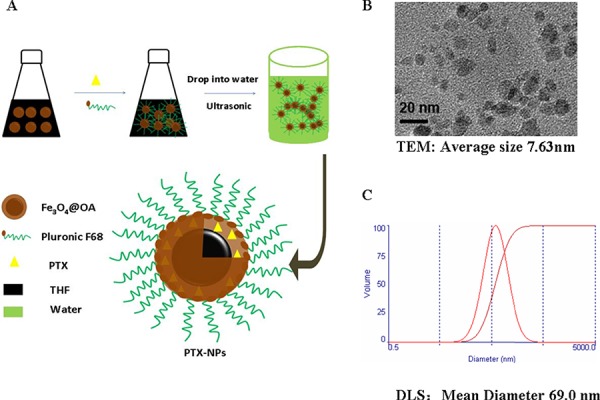
Characterization of PTX-NPs **A.** Schematic illustration of PTX-NPs preparation. PTX, oleic acid-coated iron oxide nanoparticles (Fe3O4@OA NPs) and polyoxyethylene/polyoxypropylene copolymer (Pluronic F68) were dissolved in tetrahydrofuran (THF). The mixture was added into water under sonication, and free THF in the resulting suspension was evaporated under a continuous stirring. **B.** A TEM image showing prepared nanoparticles conjugated with PTX. The average size of PTX conjugated nanoparticles is about 7.63 nm as estimated by an image analysis program based on more than 300 particles. **C.** The hydrodynamic diameter distribution of PTX-NPs was measured by dynamic light scattering (DLS).

To determine whether prepared PTX-NPs as illustrated in Fig. 2A remain a nano property, we analyzed PTX-NPs by transmission electron microscopy (TEM) and dynamic light scattering (DLS), which demonstrated that PTX-NPs have a quite uniformed core size of 7.63 nm (Fig. 2B), and hydrodynamic diameter of 69.0 nm (Fig. 2C), the latter of which includes both the core-shell and the aqueous layer.

**Figure 3:**
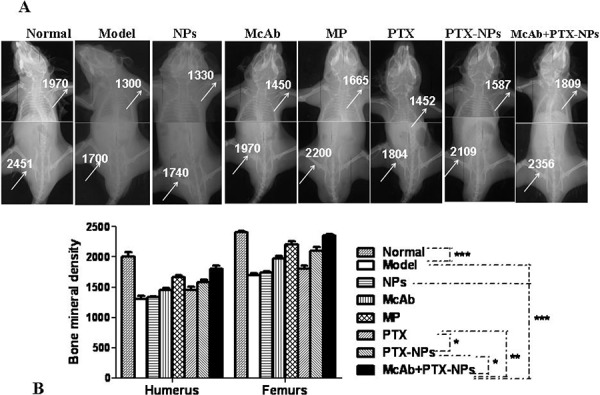
Significant improvement of BMD by McAb+PTX-NPs in MM mice **A.** Micro-CT images showing BMDs in MM mice 4 weeks after treatments with different agents as indicated. **B.** Quantification of BMD in the mice treated with different agents. The data represent mean ± SD (*n* = 6) *p < 0.05, **p < 0.01 and ***p < 0.001 referred to the differences as indicated. Bonferroni correction was applied if multiple comparisons were involved.

To evaluate the therapeutic effect of PTX-NPs on MM progression, the mice injected with MM CSCs were treated once a week with different combinations of PTX, NPs and McAb. In addition, injected mice were treated with MP, which is a common and effective regimen for MM patients. After 4 weeks of treatments, the mice were subjected to BMD examination. As shown in Fig. 3A, the lowest BMD was found with the model group in which the injected mice were treated with PBS only. The group of which the injected mice were treated with NPs alone also showed a poor BMD. In contrast, the mice treated with the other combinations showed improved BMD at different degrees. Significantly, the mice that were treated with McAb-PTX-NPs had restored BMD up to the level similar to that of the normal group and even higher than that of mice treated with MP (Fig. 3B).

**Figure 4:**
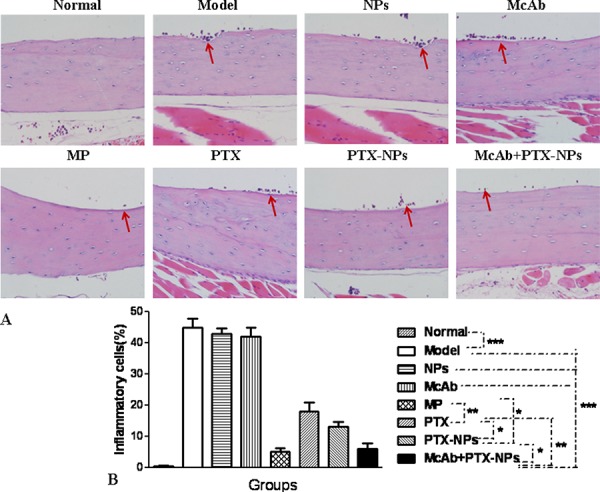
Bone lesion reduction by McAb+PTX-NPs in MM mice **A.** HE staining of femur lesions in MM mice 4 weeks after treatment with different agents as indicated (magnification, 400 ×). Arrows indicate aggregated and infiltrated inflammatory cells. **B.** Quantification of bone lesions. *p < 0.05, **p < 0.01 and ***p < 0.001, referring to the differences as indicated.

Bone lesions in treated mice were further analyzed by histology. The model mice had developed significant lytic bone lesions, including bone trabeculae destruction, sinus extension and breakage, RBC seepage and edema in the bone marrow matrix, and aggregated and infiltrated inflammatory cells (arrows) in the compact bone (Fig. 4A). The lytic bone lesion, as determined by infiltrated inflammatory cells, was markedly decreased in the mice treated with McAb-PTX-NPs compared with those treated with McAb, PTX, PTX-NPs, and model mice, respectively. However, there was no significant difference between the McAb-PTX-NP group and the MP group (Fig. 4B).

### McAb-PTX-NPs improves the kidney function of MM bearing mice

**Figure 5:**
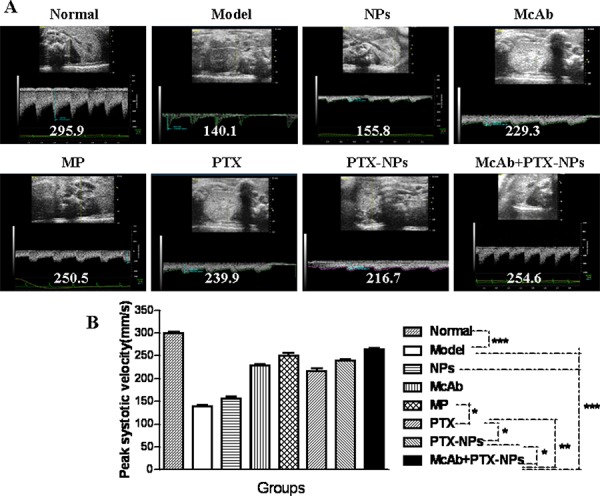
Ultrasound imaging of the BFV of renal arteries in MM mice **A.** Images showing peak systolic BFV of renal arteries in MM mice 4 weeks after treatment with different agents as indicated. **B.** Statistical analysis of peak systolic BFV of MM mice. *p < 0.05, **p < 0.01 and ***p < 0.001.

To evaluate whether McAb-PTX-NPs have any therapeutic effect on the MM-mediated kidney disorders, we analyzed the renal artery of treated mice by ultrasound imaging. As shown in Fig. 5, the renal artery of normal mice had a stable current velocity profile and a peak BFV at 295.9, whereas that of MM bearing mice without treatment had a nearly diminished current velocity wave and markedly decreased BFV (140.1). Notably, the peak BFV of the renal artery of the mice treated with McAb-PTX-NPs was significantly recovered to 254.6, which was accompanied with a stable arterial current velocity. The mice treated with MP also showed a significant recovery and had a peak BFV at 250.5, which was statistically the same as that treated with McAb-PTX-NPs. However, these mice had a less normal current velocity profile than did McAb-PTX-NPs treated mice.

**Figure 6:**
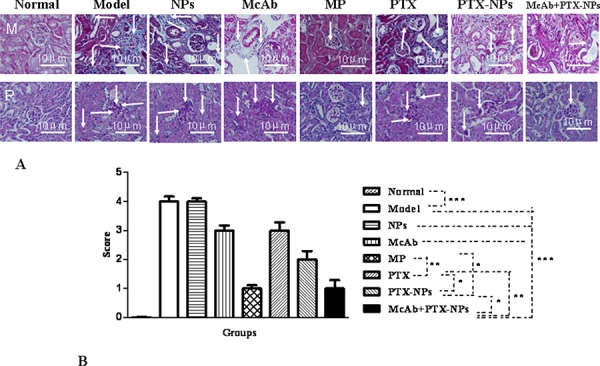
Histological analysis of kidneys of MM mice **A.** Tissue sections derived from MM mice 4 weeks after treatments with different agents were stained with either masson (M) or PAS (P) Arrows indicate representative damaged areas as described in the text. **B.** Quantitative analysis of renal damages in treated mice. *p < 0.05, **p < 0.01 and ***p < 0.001.

Renal sections derived from the mice after 28 days of treatments were also subjected to histological analysis. Apparent renal damages as indicated by large blue areas were seen in massion-stained sections from the model mice (Fig. 6A, top panel). Also, periodic acid-schiff staining revealed that the model mice had over 75% glomeruli that were apparently hardened (bottom panel), which is pathologically equivalent to a 4 score of renal damages [15, 17]. The renal damages became apparently less severe in the mice treated with the other agents. In particular, the mice treated with McAb-PTX-NPs or MP showed a significant alleviation of renal damages (Fig. 6B).

Kidney damages in MM patients are often manifested by increase in protein contents in urine and in the levels of serum free light chains (FLCs), calcium and IL-6 in serum [17–20]. To analyze the effect of McAb-PTX-NPs on these MM-associated disorders, we used ELISA to measure the contents of these soluble factors. The result of this study indicated that the treatment of MM mice with PTX-NPs significantly decreased the levels of FLC, calcium and IL-6 in serum as well as proteins in urine compared with that of PTX or McAb alone (Fig. S1). This efficiency was further enhanced by combining PTX-NPs with ABCG2 McAb. In addition to the soluble molecules, the number of RBCs, as measured by RBC counting, was also elevated similarly in the mice treated by these agents (Fig. S1).

### McAb-PTX-NPs induces strong apoptosis with BMMCs derived from MM mice

**Figure 7:**
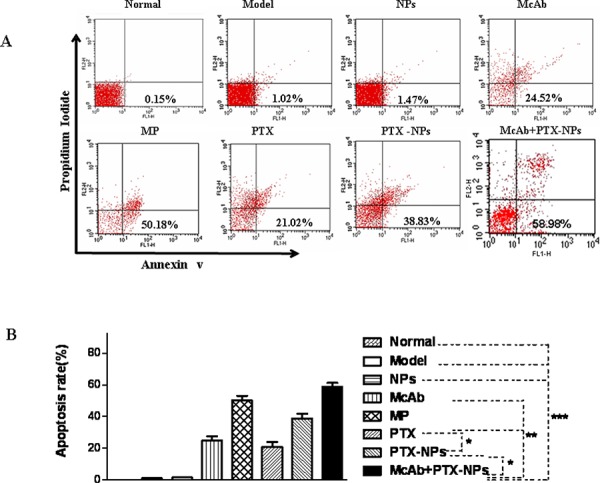
Strong apoptosis induced by McAb+PTX-NPs in BMMCs isolated from MM mice **A.** Apoptosis of BMMCs derived from treated MM mice was analyzed as described in the Method. **B.** Quantification of apoptotic BMMCs. *p < 0.05, **p < 0.01 and ***p < 0.001.

To explore a cellular mechanism for the anti-MM function of McAb-PTX-NPs, we performed flow cytometry to analyze the apoptosis with BMMCs derived from treated mice. As shown in Fig. 7A, normal and model mice or the mice treated with NPs contained few apoptotic BMMCs. In contrast, mice treated with other agents showed a marked increase in the number of apoptotic BMMCs. Among them, McAb-PTX-NPs caused the highest apoptotic rate (58.98%), which is significantly greater than those induced by PTX, or McAb or PTX-NPs alone, which ranged from 21.02% to 38.83% (Fig. 7B).

### McAb-PTX-NPs has a strong therapeutic effect on cancer cells derived from MM patients

To determine whether McAb-PTX-NPs have a therapeutic effect on primary MM cells, we isolated CD138^−^CD34^−^cells from BMMCs of three MM patients. These cells have characteristics of CSCs as determined by a series of *in vitro* assays (Fig. S2), which demonstrated that CD138^−^CD34^−^cells had a higher growth rate (Fig. S2A), a higher clonogenic potential (Fig. S2B and 2C), a higher drug resistance to vincristine (Fig. S2D), and a higher ability to form tumors in xenografted mice than did non-CD138^−^CD34^−^cells (Fig. S2E). In addition, CD138^−^CD34^−^cells from nine MM patients showed a significantly increased expression levels of ABCG2 at both mRNA and cell surface (Fig. S3).

Upon treatment with McAb-PTX-NPs, MM-derived CD138^−^CD34^−^cells underwent to significant apoptosis *in vitro* (Fig. S4). Application of McAb-PTX-NPs to NOD-SCID mice injected with 5 × 10^4^ CD138^−^CD34^−^cells increased significantly their BMD compared with the control mice (data not shown), demonstrating that McAb-PTX-NPs have also a strong therapeutic effect on primary MM cells.

## DISCUSSION

MM CSCs may be responsible for chemo-resistances, the primary cause for the clinical failure in complete elimination of MM cells. One of the possible mechanisms for drug resistance is that CSCs express high levels of ABCG2 transporter [8, 21, 22], which facilitates pumping out therapeutic drugs out of cells. Recent advances have evidenced that the targeted therapies have promised to improve the efficacy of cancer treatments by aiming at inhibition of specific molecules or signaling pathways. Thus, we hypothesized that combination of a conventional cancer drug with the ability to target ABCG2 would be a better approach to treat MM patients and may improve drug-sensitivity. In this study, we focused on CD138^−^CD34^−^cells because this phenotype cells isolated from MM cell line RPMI 8226 have the characteristics of MM CSCs, which exhibited stronger proliferation, migration, drug resistance to PTX, clone formation ability, tumorigenic potential and more ABCG2 molecular expression than the non-CD138^−^CD34^−^cells [23, 24]. In addition, we found that such population derived from MM patients possesses typical CSC features and that they are enriched in ABCG2 expression on cell surface. Our finding is essentially consistent with the previous findings by others [2, 22, 25] and by us [23, 24]. Indeed, we had previously been able to establish tumors in mice by subcutaneous injection of MM CD138^−^CD34^−^CSCs from human cell lines. However, whether these injected mice have MM characteristics remained unknown. In the presented study we injected, via dorsal tail vein, MM CD138^−^CD34^−^CSCs into NOD/SCID mice and demonstrated that these injected mice developed typical MM-associated symptoms including abnormally poor BMD, high levels of urine protein, high levels of FLC, calcium and IL-6, and impairments in the function of kidney. In view of the fact that mice normally do not contain detectable level of protein in urine and that the mice injected with CD138^−^CD34^−^CSCs exhibited a high level of urinal protein, it is likely that injected MM cells had significantly infiltrated into the kidney. Thus, the mice described here would serve as a proper murine model that reflect many pathological aspects associated with MM. Based on this model, we have been able to demonstrate that the combination of PTX-NPs with ABCG2 McAb achieved a highest therapeutic response than did any other combinations tested in this study. Importantly, McAb-PTX-NPs showed even a stronger efficacy than MP, which has a demonstrated curative effect on MM [26, 27]. Thus, combination of nano-drugs with ABCG2 McAb targeting CSCs could be ultimately developed into a better therapy for MM.

The strong therapuetic activity of McAb-PTX-NPs is apparently associated with its strong activity to induce BMMC apoptosis. The mechanisms may involve inhibition of ABCG2 and preventing of the internalized PTX from efflux out of the cells, and thereby generating a high cytotoxicity on cells. In addtion, we guess that anti-ABCG2 McAb may lyse MM cells by antibody-dependent cell-mediated toxicity and complement-dependent cytotoxicity in mice. These possible mechanisms deserve further investigations.

In conclusion, our data shows the targeted therapeutic effect of McAb-NPs-PTX on MM CD138^−^CD34^−^CSCs were demonstrated in a xenograft MM mice. To our knowledge, this study is the first to examine the efficacy of McAb-PTX-NPs therapy for MM murine model established by tail vein injection of MM CD138^−^CD34^−^cells. The findings presented provide novel insight into the use of NPs-PTX in combination with anti-ABCG2 McAb directly targeting MM CD138^−^CD34^−^CSCs and inhibiting CSC growth *in vivo*. The strategy may be an effective treatment paradigm for patients with relapsed/refractory MM.

## MATERIALS AND METHODS

### Cell line

The human MM RPMI 8226 cell line was purchased from the Cellular Institute of Basic Medical Science, Chinese Academy of Medical Sciences in Bejing, China. Cells were cultured in complete media consisting of RPMI 1640, 2 mM L-glutamine, 100 U/mL penicillin, 100 μg/mL streptomycin, and 10% fetal bovine serum at 37°C with 5% CO_2_.

### Clinical samples

Clinical bone marrow samples were obtained from 9 MM patients at stages II-III with a median age of 65 years (55–86 years) or healthy donors who granted informed consent as approved by the Nanjing First Hospital Affiliated to Nanjing Medical University. BMMCs from the MM patients were isolated by density centrifugation using Ficoll-Paque solution (Biological Chemical Factory, Shanghai, China).

### Animals

Non-obese-diabetic/severe-combined-immunodeficiency (NOD/SCID) mice at 5 weeks of age with 16 ± 2 gram in weight were purchased from Beijing Weitong Lihua Experimental Animal Technology Co., Ltd., China. All the mice were maintained in a pathogen-free facility that has a 12-hour light/dark cycle and relative humidity ranged from 40% to 50%. The enviromental temperature was maintained at 22°C and the mice were fed with steril foods. All the animal experiments were performed in compliance with the Guidelines of the Animal Research Ethics Board of Southeast University.

### Isolation of MM CD138^−^CD34^−^cells

CD138^−^cells were isolated from human MM RPMI 8226 cells or BMMCs from MM patients using mouse anti-human CD138 and anti-human CD34 microbeads (Miltenyi Biotec, Germany) followed by immune magnetic activated cell sorting (Miltenyi Biotec, Germany) as described previously [23–25, 28, 29], and the positive rate of CD138^−^CD34^−^cells checked by FACS was about 95% (data not shown).

### Establishment of a MM xenograft model

NOD/SCID mice were injected with 1 × 10^7^ MM CD138^−^CD34^−^ cells through tail veins. Eighteen days after injection, urine protein was collected by using a mouse metabolic cage and measured by enzyme-linked immunosorbent assay (ELISA). Micro-computer tomography (Micro-CT) scanning and ultrasonography were used to examine bone lesions and renal damages in mice. MM-bearing mice were randomly divided into eight groups of equal size (3 mice), including model group (mice treated with PBS), NPs group (treated with 1.2 mg/kg Fe_3_O_4_ NPs), McAb group (treated with 0.5 mg/kg anti-ABCG2 McAb), PTX group (treated with 4 mg/kg PTX), PTX-NPs group (treated with 0.122 mg/kg PTX+1.2 mg/kg NPs), McAb-PTX-NPs group (treated with 0.5 mg/kg anti-ABCG2 McAb and 0.122 mg/kg PTX+1.2 mg/kg NPs), MP group (treated with melphalan 7.5 mg/kg and prednisone 3 mg/kg) as the positive control, and normal group (no treatment). To test the anti-tumor effect of agents, 200μL of each was intravenously injected into MM-bearing mice once a week with total of four times 18 days after the mice were injected with CSCs [1, 29–31]. To analyze tumorigenicity, NOD/SCID mice were subcutaneously injected with 5 × 10^4^ CD138^−^CD34^−^cells derived from the bone marrow of MM patients, and tumors palpable at the injection sites were examined after 14 days. All the animal experiments were repeated at least twice.

### Preparation of PTX-NPs

Oleic acid-coated iron oxide NPs (Fe_3_O_4_@OA NPs) were synthesized by a two-step method to produce PTX-NPs as described previously [32]. Briefly, Fe_3_O_4_@OA NPs were suspended in tetrahydrofuran at ratio of 1:5 (w/w). The mixture was slowly added into and 1% polyoxyethylene/polyoxypropylene copolymer solution under sonication and the resulting suspension was stirred to evaporate tetrahydrofuran, and was filtered and lyophilized. The drug is loaded on PTX-NPs at a ratio of 10.71% (PTX/Fe, wt). The morphology of PTX-NPs after rehydration was observed by TEM using microscope JEM-2000EX (JEOL Ltd., Japan). The hydrodynamic diameter, size distribution and zeta potential of PTX-NPs were examined by DLS with Malvern Zetasizer 3000 (Malvern Instruments Co., H.K.).

### Analysis of bone mineral density and peak systolic blood flow velocities of renal artery

The bone mineral density (BMD) of the humerus and femurs in mice was measured by *in vivo* Micro-CT imaging with MCT-1108 (China) at a setting of voltage 45 kV and electric current 80 μA [33, 34]. The peak systolic BFV of renal artery was detected by ultrasonography with Vevo 2100 (Visualsonic, Germany). All the measurements were performed once a week with total of four times [35].

### Histological analysis of lytic bone lesions and renal impairments

After four weeks of treatment, mice were anesthetized with phenobarbital sodium and sacrificed by cervical dislocation. The humerus and femurs as well as the kidneys were harvested, and next steps were performed as described previously [1, 13].

### Measurement of serum FLC, calcium, IL-6 and urine protein and red blood cells

Serum free light chains (FLCs), calcium, cytokine IL-6 and urine protein were measured by ELISA-based kits according to the manufacturer’s protocol (eBioscience company, USA) [17, 35]. Red blood cells (RBCs) were counted as described previously [18].

### Analysis of BMMC apoptosis

5 × 10^5^ BMMCs isolated from the MM bearing mice were seeded into a 96-well plate (100μL/well), stained with FITC-conjugated Annexin V and Propidium Iodide (PI), and resuspended in PBS. The suspented cells at 100μL were incubated with 5μL Annexin V-FITC (KeyGen Biotech. Co. Ltd) and 10μL of 50 μg/mL PI for 15 min at room temperature in the dark. The stained cells were analyzed within 1 hour by flow cytometry using FACS Caliber (BD, U.S.A) [19].

### Statistical analyses

The data were plotted as mean ± SD and analyzed for statistical significance by two-tailed paired Student’s *t* test or repeated measures analysis of variance (ANOVA). *P* values less than 0.05 were considered statistically significant. Analyses were performed with the Graph Pad Prism 3.0 statistical software package (Graph Pad Company, USA).

## SUPPLEMENTARY FIGURES


