# Supplementary material for: Target therapy of multiple myeloma by PTX-NPs and ABCG2 antibody in a mouse xenograft model
Source: Oncotarget. 2015 Jul 15;6(29):27714–24. doi: 10.18632/oncotarget.4663 (PMC4695020; doi:10.18632/oncotarget.4663)
Supplement: Supplementary file 1 [file oncotarget-06-27714-s001.pdf]

SUPPLEMENTARY FIGURES

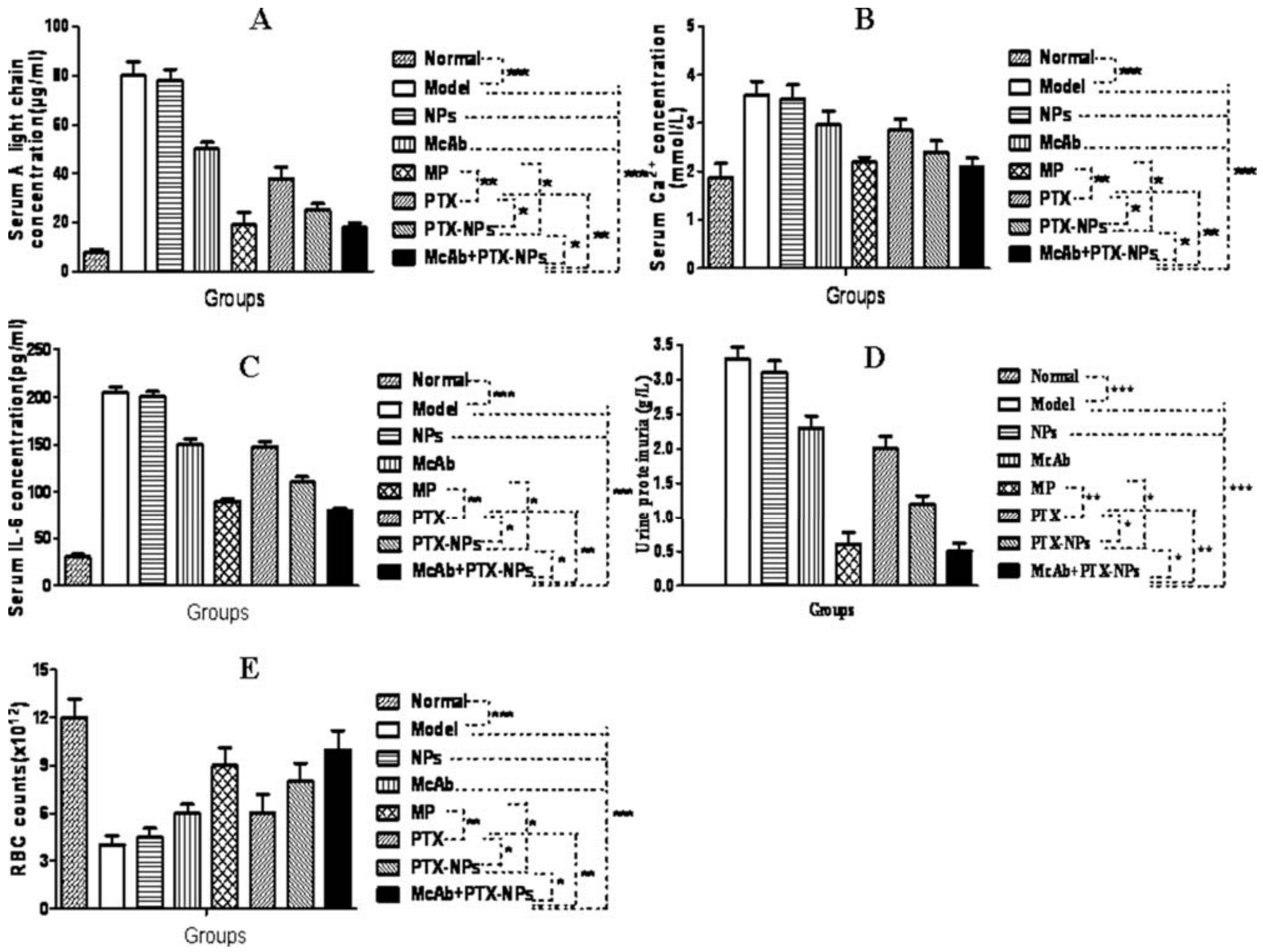

Supplementary Figure S1: Effect of McAb+PTX-NPs on FLCs, calcium, IL-6, proteinuria and RBCs in MM mice. Serum FLC **A.** serum calcium **B.** serum IL-6 **C.** proteinuria **D.** and RBCs **E.** were measured as described in the Method. All the measurements were made with MM mice 4 weeks after treatments. $^{*}p < 0.05$, $^{**}p < 0.01$ and $^{***}p < 0.001$.

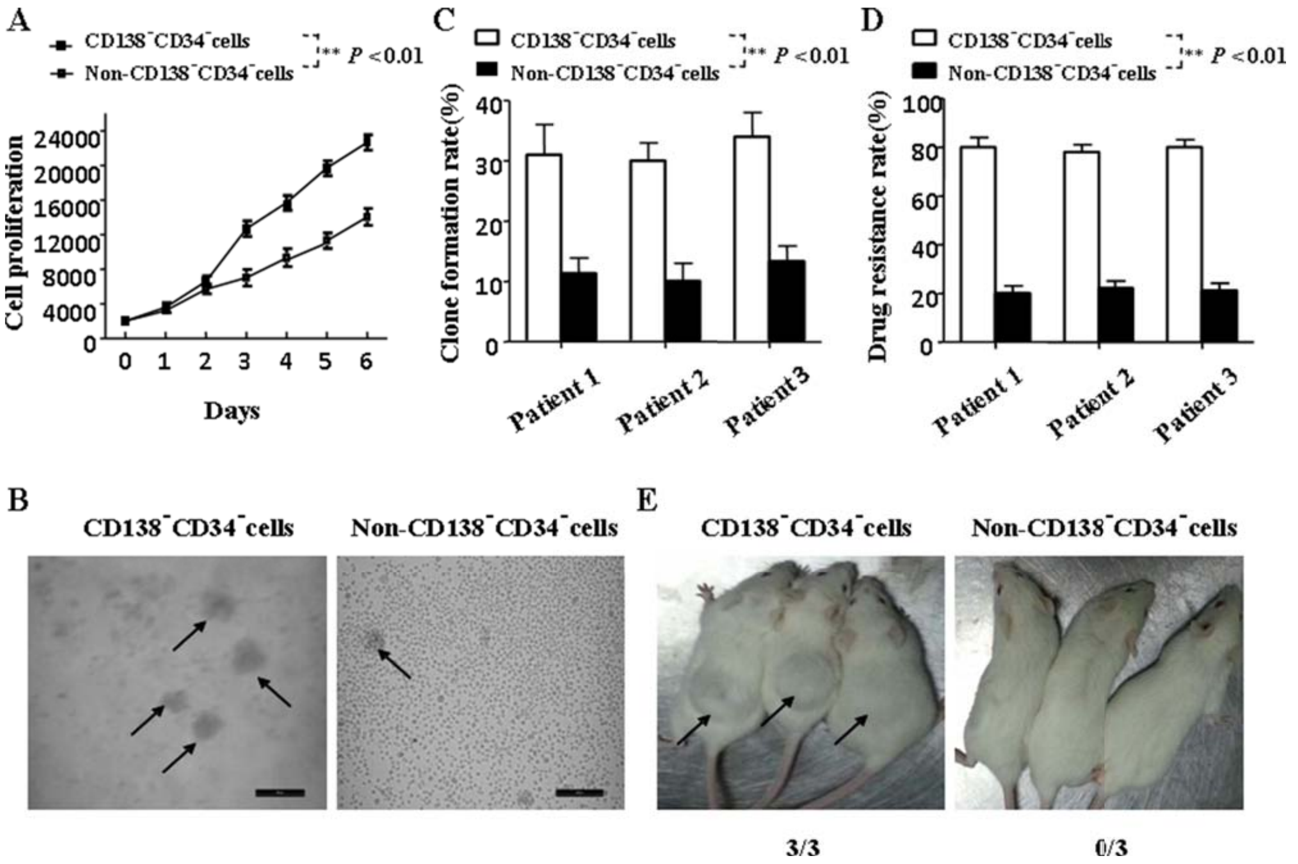

Supplementary Figure S2: Characterization of MM CD138⁻CD34⁻cells derived from MM patients. A. Proliferation of cells grown in RPMI 1640 medium; **B-C.** Clonal formation ability of cells in soft agar medium and formation rate. Bar = 100 µm. **D.** Cells were treated with 0.5 µg/mL vincristine for 72 hours, and the survival of treated cells was measured by MTT assay; **E.** Images showing tumors developed from NOD/SCID mice at 12 days after subcutaneous injection of 5×10^4 CD138⁻CD34⁻cells isolated from MM patients (references 22 and 23). Note, no tumors were found in mice injected with 5×10^4 non-CD138⁻CD34⁻cells.

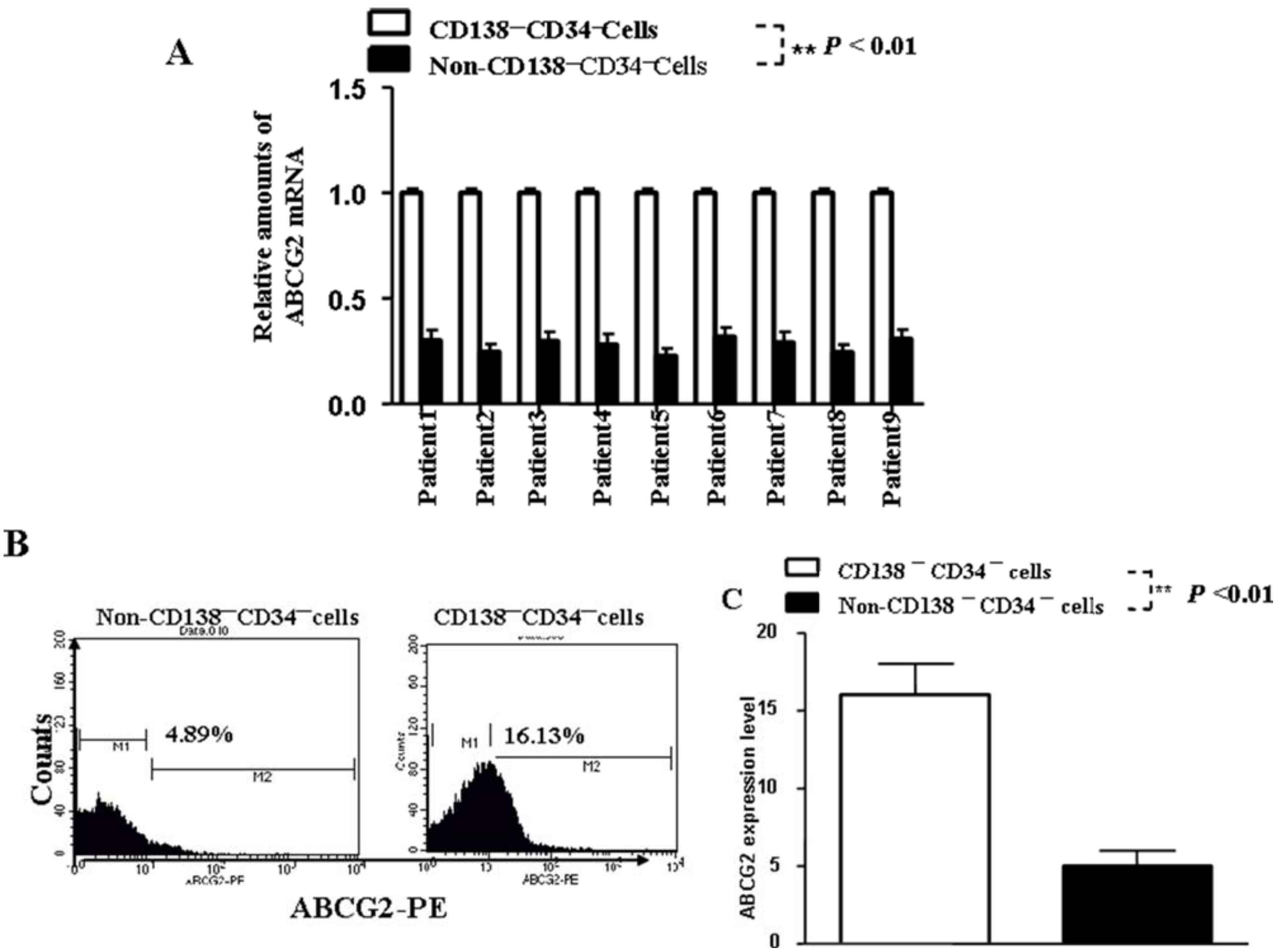

Supplementary Figure S3: Analysis of ABCG2 expression in CD138⁻CD34⁻cells and non-CD138⁻CD34⁻cells isolated from MM patients. A. ABCG2 expression was measured by quantitative PCR as described previously (references 22 and 23). **B.** ABCG2 expression was measured detected by flow cytometry. **C.** Quantification of ABCG2 expression based on flow cytometry.

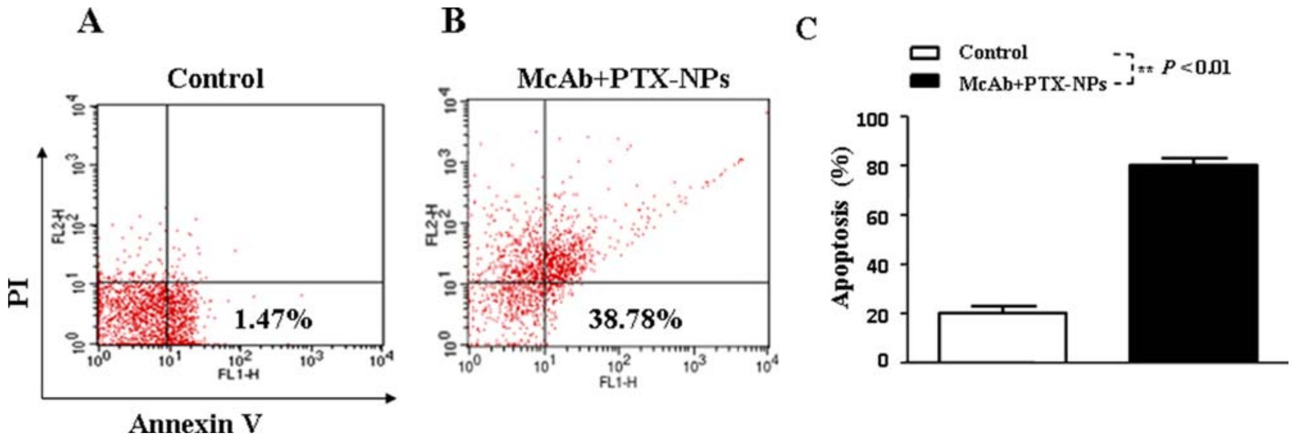

Supplementary Figure S4: Apoptosis induced by McAb+PTX-NPs in CD138⁻CD34⁻cells isolated from MM patients. MM CD138⁻CD34⁻cells were either treated with PBS **A.** or with McAb+PTX-NP **B.** Apoptosis of the treated cells was analyzed by flow cytometry(A and B) and statistical analysis **C.** n = 3.
